# Crosstalk between Long Noncoding RNAs and MicroRNAs in Health and Disease

**DOI:** 10.3390/ijms17030356

**Published:** 2016-03-11

**Authors:** Ahmed S. Bayoumi, Amer Sayed, Zuzana Broskova, Jian-Peng Teoh, James Wilson, Huabo Su, Yao-Liang Tang, Il-man Kim

**Affiliations:** 1 Vascular Biology Center, Medical College of Georgia, Augusta University, Augusta, GA 30912, USA; abayoumi@gru.edu (A.S.B.); zbroskova@gru.edu (Z.B.); jteoh@gru.edu (J.-P.T.); hsu@gru.edu (H.S.); yaotang@gru.edu (Y.-L.T.); 2 Department of Internal Medicine, Medical College of Georgia, Augusta University, Augusta, GA 30912, USA; asayed@gru.edu (A.S.); jamwilson@gru.edu (J.W.); 3 Department of Biochemistry and Molecular Biology, Medical College of Georgia, Augusta University, Augusta, GA 30912, USA

**Keywords:** cancer, chromatin, epigenetic regulation, gene regulation, heart disease, non-coding RNAs

## Abstract

Protein-coding genes account for only a small part of the human genome; in fact, the vast majority of transcripts are comprised of non-coding RNAs (ncRNAs) including long ncRNAs (lncRNAs) and small ncRNAs, microRNAs (miRs). Accumulating evidence indicates that ncRNAs could play critical roles in regulating many cellular processes which are often implicated in health and disease. For example, ncRNAs are aberrantly expressed in cancers, heart diseases, and many other diseases. LncRNAs and miRs are therefore novel and promising targets to be developed into biomarkers for diagnosis and prognosis as well as treatment options. The interaction between lncRNAs and miRs as well as its pathophysiological significance have recently been reported. Mechanistically, it is believed that lncRNAs exert “sponge-like” effects on various miRs, which subsequently inhibits miR-mediated functions. This crosstalk between two types of ncRNAs frequently contributes to the pathogenesis of the disease. In this review, we provide a summary of the recent studies highlighting the interaction between these ncRNAs and the effects of this interaction on disease pathogenesis and regulation.

## 1. Introduction

Since the discovery of non-coding RNAs (ncRNAs) in the 1960’s, the science of transcriptomics has expanded and revealed that ncRNAs participate in a wide variety of cellular processes [1]. NcRNAs have received increasing attention over the past years due to the significant roles that they play in cellular mechanisms and regulation [2,3,4]. In particular, microRNAs (miRs) and long non-coding RNAs (lncRNAs) have emerged as eminent players in human pathophysiological processes. MiRs are small RNAs comprised of 19–25 base pairs [5]. Over 2000 miRs have been identified, and their primary function has been found to regulate gene expression by either translational inhibition or promoting degradation of target messenger RNAs (mRNAs) [6]. For example, miR-1 inhibits cardiac hypertrophy both *in vitro* and *in vivo* by modulating signaling molecules of heart growth such as calmodulin [7]. On the other hand, lncRNAs are comprised of >200 base pairs and are classified into five subclasses: intergenic, intronic, sense overlapping, anti-sense, and bidirectional [8]. Each subclass is categorized by the genomic location of the lncRNA in relation to its neighboring encoding regions [9]. LncRNAs function to regulate gene expression through several diverse mechanisms: (i) They act as molecular guides and scaffolds to increase DNA interaction with proteins; (ii) They function as molecular decoys for proteins including transcription factors. Therefore, they help to effectively modulate the epigenetics by guiding chromatin-modifying complexes to the target genomic DNA loci; (iii) lncRNAs act as endogenous sponges for other types of RNAs such as mRNAs and miRs [9]. LncRNAs regulate many diseases and have been linked to a variety of biological processes [8,10]. For instance, the lncRNAs *Fendrr* (Foxf1 adjacent noncoding developmental regulatory RNA) and *Bvht* (Braveheart) have been found to be involved in heart development [11,12]. *Fendrr* is found in humans, mice, and rats and it functions to modulate the expression of multiple transcription factors (GATA-6, IRX3, FOXF1, NKX2-5, PITX2, and TBX3) that participate in the regulation of promoter regions [11]. *Bvht* regulates cardiomyocyte differentiation and works upstream from MesP1, a significant master regulator in heart cell lineage [12]. Most literatures pertaining to lncRNAs and miRs have focused on novel discoveries of their roles in human pathophysiology; however, less work has been done to elucidate the interaction of lncRNAs and miRs in health and disease. Here, we seek to summarize the current knowledge in the interaction of lncRNAs and miRs and to discuss how they work together to modulate disease outcomes. We first discuss the crosstalk between lncRNAs and miRs in cancers and cardiovascular diseases where its importance has been extensively studied. We will then describe the interaction of these two ncRNAs in other diseases in which the significance of the crosstalk is just beginning to be elucidated.

## 2. Interaction between Long Non-Coding RNAs and MicroRNAs in Cancers

NcRNAs have been found to be pivotal players in regulating a wide range of cancers [13,14,15,16]. In fact, the aberrant expression of lncRNAs, miRs and their downstream targets has been identified in multiple types of cancers ranging from bladder cancer to renal cell carcinoma (Table 1).

### 2.1. Bladder Cancer

Bladder cancer is the most common malignancy involving the urinary system. Patients with bladder cancer typically present with gross or microscopic hematuria although irritative and obstructive voiding symptoms can be the initial manifestation [33]. Unexplained hematuria requires the evaluation of the bladder and upper urinary tract in order to rule out urinary tract malignancy in individuals over the age of 40. Cystoscopy with biopsy is the gold standard for the initial diagnosis of bladder cancer. Stage of this cancer is an important factor to determine the appropriate treatment. The current treatment options include surgery, chemotherapy, or radiotherapy [34]. Several studies have recently shown that ncRNAs play significant roles in bladder tumorigenesis.

**Figure 1:**
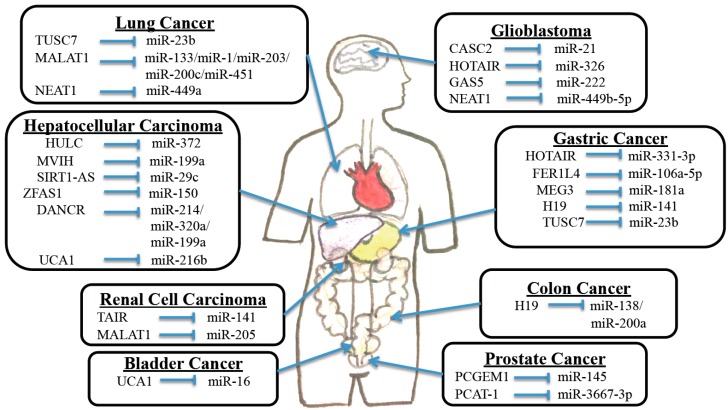
The crosstalk between lncRNAs and miRs in cancers.

Wang *et al.* found that miR-1 plays tumor suppressive roles via downregulation of the lncRNA urothelial cancer associated 1 (UCA1) in bladder cancer. The authors found that miR-1 was able to directly bind to 3′-untranslated regions (3′-UTRs) of UCA1 and effectively inhibit its expression. Both increased cell apoptosis and decreased cell motility were observed when urinary cancer cell lines were transfected with miR-1 mimic or UCA1 shRNA [35]. This study suggests that the miR-1/UCA1 axis may have potential to be developed as a therapeutic option for this cancer. A separate study found that miR-16 directly bound to the 3′-UTR of glutaminase 2 (GLS2) mRNA and regulated *GLS2* gene expression. GLS2 is a protein involved in glutamine metabolism and it is overexpressed in bladder cancer. The authors went on to show that overexpression of UCA1 and GLS2 resulted in decreased production of radical oxygen species in cancer cells, perhaps further augmenting their survival capabilities (Figure 1). Finally, they showed that UCA1 binds to miR-16 to confer the sponge effect, which may ultimately account for the increased levels of GLS2 [36].

Lastly, Han *et al.* discovered that miR-125b suppresses bladder cancer progression via inhibition of oncogene *SIRT7* and lncRNA MALAT1 (Table 1). The authors found that miR-125b and both *SIRT7* and MALAT1 are inversely expressed, with significant downregulation of this miR in urothelial cancer cells. The authors went on to prove that upregulation of miR-125b or downregulation of *SIRT7* inhibited cell proliferation and migration, while increasing apoptosis. Furthermore, MALAT1 silencing was also shown to result in decreased bladder cancer cell growth, motility, and increased apoptosis. Therefore, the interaction between miR-125b and MALAT1/*SIRT7* illustrates another example of aberrant ncRNA interactions to affect cancer cell tumorigenesis [17].

### 2.2. Breast Cancer

Breast cancer is the most frequently diagnosed cancer and the leading cause of cancer death in women. Breast cancer is treated with a multidisciplinary approach depending on the stage at the presentation. Treatment regimens involving surgical oncology, radiation oncology, and medical oncology have been associated with a reduction in breast cancer mortality. Screening is typically done through routine mammograms, while diagnosis is usually made after examining biopsy of suspicious lesions [37]. NcRNAs have also been identified as significant players in this cancer, and several studies have shown the importance of ncRNAs.

The lncRNA HOX transcript antisense intergenic RNA (HOTAIR) is overexpressed in breast cancer and associated with breast cancer progression. MiR-7 levels have been shown to be low in breast cancer samples, and it has been associated with inhibiting the epithelial to mesenchymal transition (EMT) of cells and preventing metastasis. Zhang *et al.* found that HOTAIR inhibits HOXD10, resulting in the indirect downregulation of miR-7 (Table 1). The authors went on to show that miR-7 inhibits the histone methyltransferase *SETDB1*, and can partially reverse the EMT via the STAT3 pathway [18]. Li *et al.* showed that HOTAIR was able to downregulate miR-568, which led to overexpression of one of its target genes, *NFAT5* (Table 1). *NFAT5* has been implicated in promoting EMT, invasion, and metastasis. Authors found that HOTAIR-mediated upregulation of *NFAT5* resulted in increased expression of the calcium binding protein S100A4 and vascular endothelial growth factor C (VEGF-C). These proteins are well-known to contribute to several cell properties that increase metastasis by regulation of MMPs, angiogenesis, and permeability of blood and lymph vessels [19]. These studies again indicate that downregulation of a miR-lncRNA crosstalk is responsible for the upregulation of a gene, resulting in the increased metastatic ability of cancer cells.

Interestingly, Shi *et al.* showed the effect of a lncRNA named activated by TGF-B (ATB) on trastuzumab chemotherapy resistance in breast cancer patients. ATB was found to be the most upregulated lncRNA in trastuzumab-resistant (TR) cell lines and breast cancer patient samples. This lncRNA exerts its effects on TR by direct binding to miR-200c. MiR-200c plays a tumor suppressive role by regulating the expression of both *ZEB1* and *ZNF*-*217* and subsequently preventing EMT. They found that the high levels of ATB effectively sequesters miR-200c to cause increased expression of *ZEB1* and *ZNF-217*, which ultimately increases the invasion and metastasis of cancer cells [38].

Another important study addressed the role of ncRNAs in triple-negative breast cancer (TNBC; ER-, *HER2*-, and PR-negative breast cancer). Eades *et al.* found that the lncRNA-Regulator of Reprogramming (lncRNA-RoR) functions as a competing endogenous RNA (ceRNA) sponge for miR-145 [39]. Previous studies have suggested that significant downregulation of miR-145 may be the defining characteristic in TNBC. Interestingly, lncRNA-RoR is dramatically upregulated in TNBC and metastatic disease. The authors went on to show that suppression of miR-145 resulted in upregulation of one of its target genes, *ARF6*. ARF6 is well known for contributing to the metastatic potential of cells. They concluded that miR-145 does not actually affect proliferation or apoptosis, but instead regulates tumor cell invasion via the lncRNA-RoR/miR-145/ARF6 pathway [39].

### 2.3. Gastric Cancer

Gastric cancer is the second most common cancer worldwide. While it is less common in the United States, it still accounts for a significant proportion of cancer-related deaths. There are several types of cancers that can occur in the stomach, but the most common type is adenocarcinoma. It is originated from one of the cell types found in the lining of the stomach. Most patients with gastric cancer are asymptomatic and initially present with advanced disease. Symptoms of gastric cancer are typically vague and nonspecific. Patients commonly complain of dyspepsia, abdominal pain, nausea, vomiting, anorexia, and weight loss. Diagnosis is commonly made using upper endoscopy with biopsy, CT scan, and/or PET scan. Surgery is the only definitive cure option, but it is performed in only the low percentage of the patients that are diagnosed when the disease is in its early stages [40].

Several studies have illustrated the roles of ncRNAs in this disease. Overexpression of one lncRNA, HOX transcript antisense intergenic RNA (HOTAIR), was found to possibly confer a malignant phenotype in tumor cells. It represents a biomarker associated with poor prognosis. HOTAIR exerts its effects by functioning as a sponge on miR-331-3p and subsequently blocking miR-331-3p-mediated suppression on its target gene *HER2* (Table 1 and Figure 1), a well-known regulator of gastric cancer [20].

Xia *et al.* looked at the lncRNA FER1L4 and found that it suppressed gastric cancer cell growth. The authors discovered that FER1L4 acts as a ceRNA that takes up miR-106a-5p (Table 1 and Figure 1). This in turn prevents miR-106a-5p from suppressing one of its targets, the tumor suppressor gene *PTEN* [21].

LncRNA *MEG3* was also found to be capable of inhibiting gastric cancer cell proliferation, migration, and invasion by competitively binding with members of the miR-181 family such as *MEG3* sequestering oncogenic miR-181a (Table 1 and Figure 1). The miR-181 family has been shown to exert oncogenic effects via suppression of the apoptosis gene *Bcl-2*. Therefore, the sequestration of miR-181a by *MEG3* results in upregulation of Bcl-2, and suppresses gastric carcinogenesis [22].

Tumor suppressor candidate 7 (TUSC7) is another lncRNA that is known to be a p53-regulated tumor suppressor. TUSC7 acts as a ceRNA to repress miR-23b (Figure 1). Effects of miR-23b overexpression in gastric cancer cells were found to include increased cell growth and reversal of TUSC7 overexpression-mediated decrease in cell growth. Therefore, the significant downregulation of TUSC7 in gastric cancer likely allows for the oncogenic characteristics of miR-23b to run unopposed. It was also shown that TUSC7 is a key regulatory hub in gastric cancer [41], suggesting that biomarkers or therapeutic interventions may be developed using this lncRNA.

### 2.4. Hepatocellular Carcinoma

Hepatocellular carcinoma (HCC) is an aggressive tumor that often occurs in the setting of chronic liver disease and cirrhosis. Although surgical resection is often the preferred treatment, the majority of patients are not eligible because of advanced tumor stage or underlying liver dysfunction [42]. Shi *et al.* looked at microvascular invasion in hepatocellular carcinoma (MVIH), a lncRNA aberrantly expressed in HCC, and found that it was capable of inhibiting miR-199a (Figure 1). This inhibition was subsequently linked with an increase in cell growth, an inhibition in cell apoptosis, and even associated with increased microvascular invasion into the HCC. In fact, both the luciferase reporter and RNA immunoprecipitation experiments showed that miR-199a was able to directly bind with MVIH RNA [43].

A different study identified a novel mutation in another lncRNA, SIRT1-AS that led to a decreased risk of HCC. *SIRT1* is a gene that plays an integral part in cell proliferation, apoptosis, and metabolism and has been found to be overexpressed in HCC samples. SIRT1-AS is believed to work by suppressing the miR-mediated translational repression of *SIRT1* mRNA by masking the miR-29c binding site on the SIRT1’s 3′-UTR (Figure 1). SIRT1-AS bound to SIRT1’s 3′-UTR prevented miR-29c binding and stabilized SIRT1 mRNA. This results in the increased levels of SIRT1 and subsequent increase in cell survival. The 622C mutation that was identified in SIRT1-AS effectively changes the structure of the lncRNA and prevents it from binding and stabilizing the *SIRT1* mRNA. In fact, overexpression of the 622C mutant was found to suppress HCC cell proliferation, and decrease the risk of HCC, offering another potential target for gene therapy [44].

ZFAS1 is a lncRNA that functions as an oncogene in HCC progression by binding miR-150 and abrogating its tumor-suppressive function (Figure 1). MiR-150 represses HCC cell invasion by inhibiting *ZEB1* as well as matrix metalloproteinases MMP14 and MMP16. These findings support the function of ZFAS1 in metastatic progression and suggest its candidacy as a new prognostic biomarker and target for clinical management of HCC [45].

DANCR is a lncRNA that was found to markedly increase stemness features in HCC cells, resulting in increased tumorigenesis as well as intra- and extra-hepatic tumor colonization. DANCR was found to exert its actions primarily through interaction with mRNA of the cadherin-associated protein catenin beta 1, CTNNB1. Specifically, DANCR was found to block the repressing effect of miR-214, miR-320a, and miR-199a on CTNNB1, which resulted in the upregulation of this gene (Figure 1). Interestingly, upregulation of CTNNB1 through DANCR overexpression resulted in an increase in the spheroid formation and stem cell-like features of the cancer cells. Conversely, DANCR knockdown successfully attenuated the stemness and *in vivo* interference with DANCR action, leading to decreased tumor cell survival, tumor shrinkage, and improved mouse survival [46].

The lncRNA urothelial carcinoma-associated 1 (UCA1) was also found to be significantly overexpressed in HCC. UCA1 is thought to contribute to HCC development/pathogenesis by acting as an endogenous sponge that directly binds to miR-216b and downregulates miR-216b expression (Figure 1). MiR-216b-mediated repression of FGFR1 is therefore mitigated, leading to an increased level of FGFR1 in the cancer cells. Additionally, the authors found that loss-of-function of UCA1 resulted in downregulation of ERK ½ and p-ERK ½, and inhibited growth and metastasis both *in vitro* and *in vivo*. This study suggests that UCA1 likely increases the proliferation and metastasis of HCC cells through activation of FGFR1/ERK signaling pathways [47].

HULC is another lncRNA overexpressed in HCC, and it has been linked to abnormal lipid metabolism in HCC cells. It is shown that HULC plays a role in deregulating lipid metabolism through a signaling pathway involving miR-9, PPARA, and ACSL1. Interestingly, a positive feedback loop involving cholesterol and RXRA was found to be capable of driving HULC signaling in hepatic cancer cells [48]. It is also reported that HULC may act as an endogenous sponge capable of downregulating several miRs, including miR-372 (Table 1 and Figure 1). Inhibition of miR-372 leads to reducing translational repression of its target gene, *PRKACB*. *PRKACB* is known to play a significant role in cAMP-responsive element binding protein (CREB) activation [29].

Linc00974 is another lncRNA that was found to be significantly upregulated in HCC. Tang *et al.* found a strong correlation between Linc00974 and KRT19, which is a known biomarker associated with HCC prognosis. The authors found that hypomethylation of the Linc00974 promoter led to its subsequent upregulation. They then identified that a positive correlation existed between Linc00974 and KRT19. It was then discovered that Linc00974 acts a sponge for miR-642 that has been shown to repress KRT19 levels. Again, the removal of miR-mediated suppression by a lncRNA is responsible for the increased levels of KRT19. Finally, it was found that KRT19 was strongly associated with the NOTCH and TGF-β pathways as identified by cDNA microarray analysis. *In vitro* knockdown of Linc00974 resulted in an inhibition of cell proliferation and invasion with increased apoptosis and cell cycle arrest [49]. Therefore, both Linc00974 and KRT19 may be novel indices for clinical diagnosis of tumor growth and metastasis in HCC, while Linc00974 may also become a potential therapeutic target for the prevention of HCC progression.

HOTTIP was identified as the most significantly upregulated lncRNA in human HCCs, even in early stage of HCC formation. HOTTIP is believed to confer its oncogenic effects by upregulating several neighboring *HOXA* genes. Tsang *et al.* found that miR-125b was capable of reducing HOTTIP-coupled luciferase activity and suppressed the endogenous level of HOTTIP (Table 1). Functionally, knockdown of HOTTIP attenuated HCC cell proliferation *in vitro* and markedly abrogated tumourigenicity *in vivo*. In addition, knock-down of HOTTIP also inhibited the migratory ability of HCC cells and significantly abrogated lung metastasis in an orthotopic implantation model in nude mice [28].

Lastly, a different study evaluated the role of the lncRNA *PTENP1* and its parent tumor suppressor gene *PTEN*. Loss of *PTEN* or *PTENP1* has been shown to frequently occur in many cancers including HCC. In order to further clarify the effects of *PTENP1*, a *PTENP1*-expressing sleeping beauty (SB)-based hybrid baculovirus (BV) vector was introduced into mice bearing HCC tumors. It was shown that the sustained increase in *PTENP1* effectively mitigated tumor growth, suppressed intra-tumoral cell proliferation, elicited apoptosis, induced autophagy, and inhibited angiogenesis. Furthermore, the authors found that miR-17, miR-19b, and miR-20a are involved in the suppression of multiple genes associated with increased autophagy including PHLPP, ULK1, P62, and *ATG7*. They also found that both *PTENP1* and *PTEN* can bind these miRs and acted as a miR sponge resulting in decreased levels of miR-17, miR-19b, and miR-20a. This negative regulation induced autophagy by removing the miR-mediated suppression [50].

### 2.5. Prostate Cancer

Prostate cancer is the second most common cancer in men worldwide. A high percentage of patients present with local or locoregional disease due to the widespread use of prostate cancer screening (PSA and/or rectal exam). Some patients with advanced disease present with metastasis-related symptoms including urinary tract obstruction, hematuria, and bone pain. The decision to pursue treatment depends on the stage at presentation and projected life expectancy of the patient. Current treatment options revolve around surgery, chemotherapy, hormonal therapy, or radiation [51]. Several studies have begun to elucidate the roles of ncRNAs in this form of cancer.

Chiyomaru *et al.* used lncRNA profiling analyses to show that genistein treatment significantly decreased the level of HOTAIR in prostate cancer cell lines. Of note, genistein is a soy isoflavone that has antitumor activity both *in vitro* and *in vivo*. Genistein exhibits its antitumor effects by regulating several cell signaling pathways (WNT, *AKT*, JAK/STAT) and miRs. Similarly as in gastric cancer, HOTAIR expression was found to be higher in castration-resistant prostate cancer cell lines than in normal prostate cells. The authors also found that miR-34a was able to directly bind to HOTAIR and decrease its expression. They also demonstrated that genistein treatment upregulated miR-34a, subsequently mitigating the oncogenic effects of HOTAIR. In fact, knockdown of HOTAIR decreased prostate cancer cell proliferation, migration, invasion, and induced apoptosis and cell cycle arrest [52].

Another group described the role of the oncogenic lncRNA PCAT-1 in prostate cancer cell proliferation through interaction with *cMyc*. Using a luciferase assay, the authors found that PCAT-1 was able to increase *cMyc* expression by binding to the 3′-UTR of *cMyc*. The authors went on to show that PCAT-1-mediated proliferation is dependent on *cMyc* protein stabilization. Additionally, they determined that miR-34 family and miR-3667-3p were capable of binding to *cMyc* and PCAT-1 (Table 1 and Figure 1). In fact, they found that PCAT-1 exerted a protective effect on *cMyc* expression when miR-34a was overexpressed, and miR-3667-3p specifically bound to PCAT-1 in a *cMyc*-independent manner. Functional analyses proved that miR-34a and miR-3667-3p decreased cell proliferation via the PCAT-1/*cMyc* pathway [30].

Lastly, another lncRNA, prostate cancer gene expression marker 1 (PCGEM1) is also overexpressed in prostate cancer, and associated with reduction in chemotherapy-induced apoptosis. MiR-145 has been shown to function as a tumor suppressor in prostate cancer. It was found that PCGEM1 directly bound miR-145 and resulted in reciprocal regulation (Figure 1). Furthermore, functional analyses revealed that miR-145 overexpression and PCGEM1 knockdown inhibited tumor cell proliferation, migration, invasion, and induced early apoptosis [53].

### 2.6. Colon Cancer

Colorectal cancer (CRC) is a common and lethal disease. Despite substantial progress in screening and treatment, it remains the third most common cause of cancer death in the United States [54]. The majority of patients with early stage colon cancer are diagnosed via screening colonoscopy, but microcytic anemia, constipation, and melena are common symptoms that present in patients with undiagnosed disease [55]. It has recently been shown that ncRNAs play critical roles in the pathophysiology of CRC. Liu *et al.* illustrated that a lncRNA Loc285194 (LSAMP antisense RNA 3) exhibits an inhibitory effect on miR-211, and it also acts as both an endogenous competitor and a negative regulator of p53 in colon cancer. As a master regulator for gene expression, *p53* is able to directly or indirectly regulate numerous protein-coding and non-coding genes, including miR-211. This study suggests that loc285194 is a p53-regulated tumor suppressor, which acts in part through the repression of miR-211 and subsequently helps to repress colon cancer [56].

Another lncRNA, H19 has recently been identified as an oncogene aberrantly expressed in multiple cancer types. In fact, elevated levels of H19 have been tightly linked to colorectal, ovarian, and gastric cancers. H19 has been found to be highly expressed in mesenchymal-like cancer cells and is believed to promote epithelial to mesenchymal transition by functioning as a miR sponge that primarily affects miR-138 and miR-200a [57]. Therefore, increased levels of H19 are believed to minimize the repressive function of these miRs and limit their regulatory roles in colorectal cancer. Ultimately, the development of novel treatment modalities targeting these lncRNAs may offer a powerful adjuvant to therapy in the quest to cure this common and deadly disease.

### 2.7. Renal Cell Carcinoma

Renal cell carcinoma (RCC) originates within the renal cortex and constitutes a high percent of primary renal neoplasms. The classic triad of RCC symptoms includes flank pain, hematuria, and a palpable abdominal renal mass. However, this constellation of symptoms occurs in a low percentage of patients and is suggestive of advanced disease. While CT or MRI imaging can sometimes identify the disease, biopsy is required for confirmation. Treatment of RCC depends on whether the disease is clinically localized or advanced at initial presentation [58]. Treatment typically consists of surgery with or without chemotherapy and is used for both curative or palliative purposes [59]. Recently, many studies have implicated lncRNAs as gene regulators and prognostic markers in RCC.

Hirata *et al.* looked at lncRNA MALAT1 in RCC with a specific focus on its transcriptional regulation and its interactions with *Ezh2* and miR-205 (Table 1 and Figure 1). The authors found that MALAT1 expression was higher in human RCC tissues, where it was associated with reduced patient survival. They went on to show that MALAT1 expression was reciprocally correlated with miR-205, a tumor suppressing miR that is downregulated in RCC. They also identified a positive correlation between MALAT1 and the transcription factor c-Fos. Functionally, MALAT1 silencing decreased RCC cell proliferation and invasion and increased apoptosis [31]. These findings illuminate how overexpression of MALAT1 confers an oncogenic function in RCC, and may offer a novel therapeutic target for this disease.

### 2.8. Lung Cancer

Although lung cancer remains the leading cause of cancer mortality in both men and women in the United States, advances in detection and treatment have increased the likelihood of long-term survival. The majority of patients with lung cancer have advanced disease at clinical presentation. Common manifestations of the disease include cough, hemoptysis, dyspnea, weight loss, and chest pain. Treatment is typically dictated by the stage of the cancer at the time of detection. Treatment options including surgical resection, chemotherapy such as EGFR inhibitors, and radiation are typically employed depending on the characteristics of the tumor [60]. Multiple studies have shown that ncRNAs also play roles in lung cancer.

You *et al.* looked at the interaction between the lncRNA, nuclear enriched abundant transcript 1 (NEAT1) and miR-449a in lung cancer cells (Figure 1). MiR-449a was shown to function as a tumor suppressor in lung cancer by decreasing proliferation and increasing apoptosis. Additionally, miR-449a overexpression affected the cell cycle by lengthening the G1/G0 phase and shortening the S and G2/M phases. It is also known that miR-449a expression was negatively correlated with NEAT1 expression [61].

## 3. Interaction between Long Noncoding RNAs and MicroRNAs in Cardiovascular Diseases

**Figure 2:**
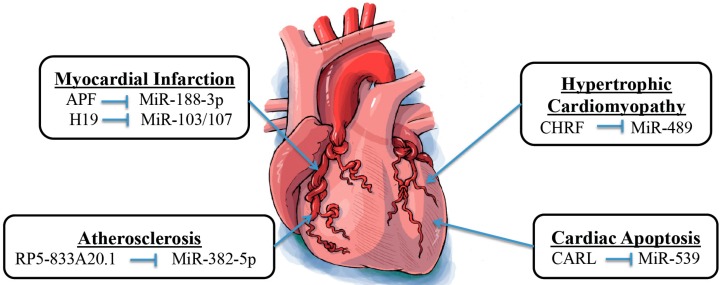
The crosstalk between lncRNAs and miRs in heart diseases.

Both lncRNAs and miRs have been discovered to play important regulatory roles in many pathophysiological processes. Moreover, aberrant expression of lncRNAs and miRs has been associated with a variety of diseases, including cardiovascular abnormalities (Table 2 and Figure 2). The pathological function of these ncRNAs in the cardiovascular system has extensively been studied, and new discoveries are gradually revealing their importance. Understanding the interaction between lncRNAs and miRs is fundamental to provide further insights into the mechanism of various cardiovascular diseases.

### 3.1. Atherosclerosis

Atherosclerosis is a progressive vascular disease caused by the development of cholesterol/calcific plaques on the inner vascular wall in response to loss of endothelium. Multiple factors such as smoking and high cholesterol diet can predispose people to develop atherosclerosis [69,70]. *Nuclear factor IA* (*NFIA*) is a well-known gene that regulates cholesterol homeostasis in the body [71]. It was shown that lentivirus-mediated overexpression of NFIA in apolipoprotein E–deficient mice increased circulation of high-density lipoprotein, reduced circulation of low-density lipoprotein and very-low-density lipoprotein, decreased circulation of inflammatory cytokines including interleukin-1β, interleukin-6, tumor necrosis factor-α, and C-reactive protein, enhanced reverse cholesterol transport, and promoted regression of atherosclerosis [72]. Moreover, Hu *et al.* reported that lncRNA RP5-833A20.1 may regulate *NFIA* expression by modulating miR-382-5p expression *in vitro* (Table 2 and Figure 2). Based on multiple lines of evidence, authors ultimately concluded that the RP5-833A20.1/miR-382-5p/*NFIA* pathway is essential for the regulation of cholesterol homeostasis and inflammatory responses [62]. This newly identified pathway also offers new therapeutic targets for preventing or delaying the progression of atherosclerosis. It was also shown in a study by Tang *et al.* that the lncRNA MALAT1 can have a protective effect against endothelial dysfunction induced by ox-LDL in part by competing with miR-22-3p as an endogenous RNA [64].

### 3.2. Mitochondrial Homeostasis-Related Cardiac Apoptosis

Abnormal mitochondrial fission plays a role in the pathogenesis of many diseases, including cardiovascular diseases by disrupting physiologic mitochondrial homeostasis [73]. Modulation of mitochondrial dynamics is therefore a promising therapeutic target for treatment of cardiovascular diseases [74]. A study by Wang *et al.* showed that a lncRNA, cardiac apoptosis-related lncRNA (CARL) can suppress mitochondrial fission and apoptosis by targeting miR-539 and Prohibitin 2 (*PHB2*) (Table 2 and Figure 2). They found that *PHB2* is able to inhibit mitochondrial fission and apoptosis. They also showed that miR-539 is capable of binding to the *PHB2*’s 3′-UTR, leading to gene suppression. Furthermore, they showed that CARL acts as an endogenous miR-539 sponge, which ultimately leads to increased *PHB2* expression with subsequently decreased mitochondrial fission and apoptosis [4].

### 3.3. Cardiac Hypertrophy

Cardiac hypertrophy is an adaptive physiologic response of the heart to cardiac overload. However, with persistent overload, maladaptive cardiac hypertrophy may occur and ultimately lead to heart failure or even sudden death [75]. Thus, maladaptive cardiac hypertrophy presents as an ideal therapeutic target to stop the progression to heart failure. *MYD88* is a gene known to be overexpressed in cardiac hypertrophy. Wang *et al.* used microarray analyses to show that angiotensin II treatment reduced the levels of miR-489 in patients with cardiac hypertrophy (Table 2 and Figure 2). Moreover, they showed that *MYD88* is a direct target of miR-489 in cardiac hypertrophy. Furthermore, they identified a lncRNA named cardiac hypertrophy related factor (CHRF) that acts as an endogenous sponge of miR-489 and thus downregulates its expression [63]. Therefore, one can anticipate a therapeutic regimen for maladaptive cardiac hypertrophy that works by modulating the levels of miR-489 and CHRF.

### 3.4. Myocardial Infarction

Myocardial infarction (MI) is defined as death of cardiomyocytes secondary to a prolonged ischemic event, which typically results from an inadequate supply of oxygen to meet the heart’s metabolic demands [76,77]. MI is a major manifestation of cardiovascular diseases and is a significant cause of morbidity and mortality in the USA. Aberrant expression of several miRs has been shown to be associated with MI. For example, miR-1, miR-133, miR-208a/b, miR-499, and miR-328 have all been shown to modulate the cardiac damage following an acute MI [78,79,80]. Multiple lncRNAs such as aHIF, ANRIL, KCNQ1OT1, MIAT, and MALAT1 also have been found to be dysregulated in MI [81]. Although many studies have shown that a number of miRs and lncRNAs are involved with MI, a few studies have identified the specific interactions between miRs and lncRNAs that occur in association with MI. Wang *et al.* revealed that miR-188-3p can suppress autophagy and MI damage through its target on *ATG7*, an enzyme known to be a key player in the autophagy pathway [82]. Furthermore, the lncRNA autophagy-promoting factor (APF) has been shown to modulate autophagic cell death and MI through its interaction with miR-188-3p (Table 2 and Figure 2). APF directly inhibits miR-188-3p and leads to increased *ATG7* expression, which results in increased autophagy and infarct size [65].

Wang *et al.* also demonstrated the involvement of miR-103/107 and lncRNA H19 in myocardial necrosis through Fas-associated protein with death domain (*FADD*) [66]. Previous studies have indicated that receptor-interacting serine/threonine-protein kinase (RIPK) 1 and 3 activation can regulate specific types of necrosis. *FADD* has been shown as a negative modulator of RIPK 1 and 3 [83,84,85]. H19 acts as an endogenous sponge of miR-103/107, which results in miR-103/107 downregulation (Table 2 and Figure 2). Decreased levels of miR-103/107 prevent the downregulation of *FADD*, thus allowing *FADD* to inhibit H_2_O_2_-induced necrotic cell death by its negative regulation on RIPK 1 and 3. Researchers have only just begun to learn the importance of the interaction of ncRNAs and its significance during pathogenesis of MI. Further investigation is warranted to expand our knowledge on these ncRNAs in the hope of offering increased protection to individuals suffering from MIs.

## 4. Interaction between Long Noncoding RNAs and MicroRNAs in Other Diseases

### 4.1. Idiopathic Pulmonary Fibrosis

Idiopathic pulmonary fibrosis (IPF) is a specific form of chronic, progressive, fibrosing interstitial pneumonia of unknown cause that typically occurs in adults. Sufficient clinical evidence proving that treatment improves survival or quality of life for affected patients is lacking, and prognosis is generally poor [86]. Several studies have demonstrated that ncRNAs may play significant roles in the pathogenesis of IPF.

Previous studies have found that miR-21/31/101/29/199/let-7d are involved in the pathogenesis of IPF. Huang *et al.* used the NONCODE database to identify 34 lncRNAs with potential binding sites to these miRs. They found that of the 34 lncRNAs, nine were dysregulated in IPF samples. Four of them showed an inverse correlation with miR expression in IPF. Further studies revealed that silencing the lncRNA CD99 molecule pseudogene 1 (*CD99P1*) inhibited proliferation and the expression of α-smooth muscle actin in lung fibroblasts. Knockdown of another lncRNA that was identified, n341773 led to increased collagen expression in lung fibroblasts [87]. These results suggest that lncRNA CD99P1 and n341773 may be involved in the regulation of lung fibroblast proliferation and differentiation in IPF. Additional studies are still required to further clarify the roles of lncRNAs and miRs in IPF and to pursue them for future therapy.

### 4.2. Inflammation

Chen *et al.* looked at the roles of ncRNAs in inflammation by identifying and characterizing the interaction between PU.1/lnc-MC and miR-199a-5p. PU.1 is a hematopoietic-specific transcription factor that is involved in the regulation of lineage specific gene expression. Lnc-MC was shown to facilitate the process of monocyte/macrophage differentiation, while miR-199a-5p was shown to impair differentiation. Authors went on to discover that PU.1 positively modulates lnc-MC expression during the differentiation process. Furthermore, they found that lnc-MC was able to downregulate miR-199a-5p, which resulted in increased activin A receptor type IB (ACVR1B) expression. *ACVR1B* is another gene involved in cell growth and differentiation and is a member of the TGF-B superfamily [88]. This study revealed a novel regulatory mechanism that emphasized the role of ncRNA interaction in monocyte/macrophage differentiation.

### 4.3. Neurodegeneration

Neurodegeneration is a general term describing the progressive loss of structure or function of neurons, including death of neurons. Many neurodegenerative diseases including amyotrophic lateral sclerosis, spinocerebellar ataxia, Parkinson’s, Alzheimer’s, and Huntington’s occur as a result of neurodegenerative processes. Such diseases are currently incurable and result in progressive degeneration and/or death of the neurons [89]. NcRNAs also play roles in these progressive and ultimately terminal diseases.

Spinocerebellar ataxia type 7 (SCA7) is a neurodegenerative disorder caused by a CAG-repeat expansion in *ATXN7*, which encodes an essential component of the mammalian transcription coactivator complex, STAGA. It was found that the lncRNA-SCA7 had a positive correlation with *ATXN7* expression. Furthermore, it was shown that the correlation was dependent upon the presence of miR-124 (Table 1). It was also shown that STAGA is required for the transcription initiation of miR-124. MiR-124 in turn mediates the post-transcriptional cross-talk between lncRNA-SCA7 and *ATXN7* mRNA. In the disease, mutations in *ATXN7* disrupt these regulatory interactions and result in a neuron-specific increase in *ATXN7* expression. This increase was found to be most prominent in SCA7 disease-relevant tissues, namely the retina and cerebellum. Interestingly, miR-124 expression is known to be significantly enriched in these two tissue types [32]. These results illustrate how ncRNA-mediated feedback regulation of a ubiquitously expressed housekeeping gene may contribute to specific neurodegeneration.

## 5. Conclusions

In conclusion, our exploration of the current state of knowledge showed that miRs and lncRNAs are not only individually important in the regulation of disease, but that their cross communication represents a new aspect of disease pathogenesis and progression. The involvement of ncRNA interaction in disease regulation is not confined to a subset of diseases or organs, but has been shown to direct the pathogenesis of a wide range of critical diseases affecting multiple organ systems. This has turned the focus of researchers and clinicians interested in ncRNAs towards their potential development into disease biomarkers and therapeutics. Not only can they act as powerful biomarkers for early disease detection, but might also be exploited even further to be utilized as therapeutic agents.

## Figures and Tables

**Table 1:** Identification of long noncoding RNA-microRNA-target gene axis in cancers and neurodegeneration.

Disease	LncRNA	MiRs	Target Genes	Reference
Bladder Cancer	MALAT1	MiR-125b	*SIRT7*	[17]
Breast Cancer	HOTAIR	MiR-7	*SETDB1*	[18]
	HOTAIR	MiR-568	*NFAT5*	[19]
Gastric Cancer	HOTAIR	MiR-331-3p	*HER2*	[20]
	FER1L4	MiR-106a-5p	*PTEN*	[21]
	MEG3	MiR-181a	*MEG3*	[22]
	H19	MiR-141	*ZEB1*	[23]
	ANRIL	MiR-99a/449a	*MTOR/CDK6/E2F1*	[24]
	H19	MiR-675	*RUNX1*	[25]
Glioma	HOTAIR	MiR-326	*FGF1*	[26]
	GAS5	MiR-222	*GAS5*	[27]
Hepatocellular Carcinoma	HOTTIP	MiR-125b	*HOXA*	[28]
	HULC	MiR-372	*PRKACB*	[29]
Prostate Cancer	PCAT-1	MiR-3667-3p	*cMYC*	[30]
Renal Cell Carcinoma	MALAT1	MiR-205	*EZH2*	[31]
Neurodegeneration	SCA7	MiR-124	*ATXN7*	[32]

**Table 2:** Identification of long noncoding RNA-microRNA-target gene axis in cardiovascular diseases.

Disease	LncRNA	MiRs	Target Genes	References
Atherosclerosis	RP5-833A20.1	MiR-382-5p	*NFIA*	[62]
Cardiac Apoptosis	CARL	MiR-539	*PHB2*	[4]
Cardiac Hypertrophy	CHRF	MiR-489	*MYD88*	[63]
Endothelial Dysfunction	MALAT1	MiR-22-3p	*CXCR2/AKT*	[64]
Myocardial Infarction	APF H19	MiR-188-3p MiR-103/107	*ATG7* *FADD*	[65] [66]
Ventricular Septal Defect	MALAT 1	MiR-133	*SRF*	[67,68]
